# The changes in health-related quality of life after attending cardiac rehabilitation: A qualitative systematic review of the perspective of patients living with heart disease

**DOI:** 10.1371/journal.pone.0313612

**Published:** 2025-01-30

**Authors:** Amineh Rashidi, Lisa Whitehead, Helena Halton, Lisa Munro, Ian Jones, Lisa Newson

**Affiliations:** 1 School of Nursing and Midwifery, Edith Cowan University Joondalup Campus, Joondalup, Australia; 2 Nursing, TBHI: Telehealth.org; 3 Faculty of Health, Liverpool John Moores University, Liverpool, England, United Kingdom; University of the Witwatersrand Johannesburg, SOUTH AFRICA

## Abstract

**Background:**

Although the benefits of engaging in cardiac rehabilitation are well established, patient perceptions of the changes in their health-related quality of life are poorly documented. This systematic review synthesized qualitative studies on patients’ perspectives of change in their health-related quality of life after attending cardiac rehabilitation.

**Objective:**

To identify and synthesize the best available evidence on the perspective of patients living with heart disease about the changes in their health-related quality of life after attending cardiac rehabilitation.

**Methods:**

Eight databases were used to identify relevant papers published in English and peer-reviewed, and no date restrictions were considered for the search. This systematic review followed the Preferred Reporting Items for Systematic Review and Meta-Analyses (PRISMA) guidelines. The Joanna Briggs Institute (JBI) Critical Appraisal Checklist was used to appraise the quality of each paper, and two independent reviewers conducted it. A meta-aggregation approach was used to synthesize the findings of the included studies.

**Results:**

The search identified 10813 titles. Thirty-five full-text papers were reviewed after duplicates were removed, and the titles and abstracts were reviewed. Thirteen papers were retained for data synthesis. The synthesized findings were divided into three categories: building healthier habits, peer interaction, and improving mental health.

**Conclusion:**

This review demonstrates the positive changes in health-related quality of life for those patients engaged in cardiac rehabilitation. Peer interaction with other patients improved both mental and physical health. This review indicated that adopting healthier habits, including healthy eating and regular physical activity, had substantial benefits in formulating healthy behavior. The role of peers in supporting the development of a healthy lifestyle appears to be an understudied avenue and has potential for development.

## Introduction

Heart disease is an umbrella term that covers a variety of conditions, including blood vessel diseases, structural abnormalities, rhythm problems, and heart failure [1, 2]. Ischemic/coronary heart disease, the most common, is the leading cause of mortality, with nine million deaths globally each year [3, 4]. The burden of heart disease is often assessed by measuring health-related quality of life (HRQL). According to the WHO, HRQL refers to physical and psychological factors, social functioning, and well-being [5]. Poor HRQL is often experienced after cardiac events [6]. Loss of HRQL and physical functioning were reported before and after heart valve surgery [7] and reduced HRQL following myocardial infarction [8]. Cardiac rehabilitation (CR) is a secondary prevention program to improve HRQL [9]. CR is defined by the World Health Organization (WHO) as “*the sum of activities required to ensure them [patients] the best possible physical*, *mental and social conditions*, *so that they may*, *by their own efforts*, *resume and maintain as normal a place as possible in the community*” [10]. CR is an intervention that aims to improve functional capacity, healthy behavior, lifestyle, well-being and HRQL of patients with heart disease [11]. In other words, the goal of CR is to improve physical and mental conditions and prevent disability by improving healthy behavior [12, 13], which subsequently could improve HRQL [14].

CR programs offer a range of services, including exercise, dietary changes, pharmacological optimization, social and psychological well-being and educational path that increases the patient’s understanding of their illness [15]. The benefits of undertaking a CR program are well demonstrated through the reduction in disease progression, reducing hospital readmissions, and achieving a healthier outcome and a better HRQL [15–17]. Exercise is one of the core components of CR, which has been shown to improve physical outcomes, reduce physical limitations, increase physical fitness and improvements in strength [13, 18]. Managing psychological health is one of the main components of CR, which has a significant benefit in managing mental health in CR patients [19, 20]. Significant benefits have been observed in social functioning, primarily through peer-to-peer interactions [21].

Given the importance of participating in CR, which has been shown to improve HRQL [15], exploring changes in HRQL following CR can potentially improve patient outcomes. Understanding the changes in HRQL can inform the development strategies to increase the uptake of CR [22]. Furthermore, to our knowledge, the perceptions of patients with heart disease about the changes in their HRQL following attendance CR have not been undertaken. An earlier systematic review assessed the effects of CR interventions on patients’ quality of life with ischemic/coronary heart disease, not specific to HRQL, and only searched the articles published from 1999 to 2010 [23]. Other quantitative systematic reviews assessed the efficacy of providing the main element of CR on HRQL domains among patients with coronary heart disease [14]. They evaluated the HRQL benefits of exercise-based CR in Acute coronary syndrome patients [24]. Another review focused on the effects of eHealth CR on the health outcomes of patients with coronary diseases, not HRQL [25]. No review has synthesized qualitative findings to date to explore the perceptions of patients with heart disease regarding changes in their HRQL following CR. Therefore, this review aimed to synthesize the qualitative studies on patients’ perspectives of changes in their HRQL after attending CR.

## Methods

The Joanna Briggs approach to the conduct of systematic reviews was used to guide the review [26]. This systematic review was reported according to the Preferred Reporting Items for Systematic Reviews and Meta‐Analyses: The PRISMA Statement [26] (S1 File). Also, a review protocol was prepared to guide the review process, which was registered in PROSPERO CRD42022351118 [27]. The Edith Cowan University Human Research Ethics Committee assessed a proposal for the systematic review. A data management plan (2022-03975-RASHIDI) was approved and monitored as part of this procedure. Raw data was extracted from the published manuscripts, and the authors could not identify individual participants during or after this process.

### Search strategy

Eight databases were searched: MEDLINE, CINAHL, PsycINFO, Cochrane, EMBASE, Web of Science, Scopus, and Joanna Briggs Institute, used to identify relevant papers published in English and peer-reviewed journals. Databases were searched from date of inception through to September 2023. The analytical process and interpretation of data was completed by October 2023. The keywords cardiac rehabilitation, heart disease, health-related quality of life, and perceptions were used. The keywords were used as MeSH terms and amended for each database. A subject librarian supported the development of the search strategies (S2 File).

### Study selection and inclusion criteria

The articles identified in the searches were imported to EndNote, and duplicate records were removed. Two independent reviewers screened the titles and abstracts of the full text for their relevance against the eligibility criteria. The review included outpatient and inpatient rehabilitation programs and telephone and home-based CR studies. This review included all qualitative study designs, including phenomenology, grounded theory, ethnography action research, qualitative description, or mixed methods with qualitative data. This review included studies based on PICo (Population, Intervention, Comparison, Outcome). Population: adults aged 18 years and over with a diagnosis of heart disease. The diagnosis of heart diseases includes acute coronary syndrome, arrhythmia, heart failure, implantable cardioverter-defibrillator (ICD), vascular disease, and post-cardiac surgery (bypass graft and valve surgery); Phenomena of Interest: HRQL; Context: CR setting including outpatient and inpatients rehabilitation programs, telephone rehabilitation and home-based CR. Studies published in English were considered, and no date restrictions were implemented for the search. Studies excluded from this review focused on healthcare provider perceptions and rehabilitation related to stroke and quantitative studies.

### Quality appraisal and data extraction

The articles were appraised by two reviewers independently using the Joanna Briggs Institute (JBI) Qualitative Assessment and Review Instrument [28]. Any disagreements with the reviewer were resolved by involving a third reviewer to reach a consensus. No studies were excluded based on quality appraisal. The JBI standardized data extraction tools were used to extract data from the included studies [29]. The extracted qualitative data included phenomena of interest relevant to objectives, population, context, culture, location, data collection and analysis methods, and key findings.

### Data synthesis

The JBI approach, meta-aggregation, was used to synthesize the findings of the included studies [30, 31]. This approach engages aggregation or synthesis of findings to generate a set of categories that represent aggregation [30, 31]. Three levels of evidence, ‘unequivocal,’ ‘credible’, or ‘not supported,’ were considered to examine the credibility of the extracted findings [30, 31]. “*Unequivocal*: *findings accompanied by an illustrations beyond a reasonable doubt*, *therefore not open to challenge*, *credible*: *findings accompanied by the illustrations that are plausible and inferred from the date*, *therefore open to challenge and unsupported*: *findings not supported by the data*” [30, p.4]. Findings presented with more than one quotation had the highest level of credibility, while findings that did not offer quotations were omitted from further analysis. Subsequently, the primary reviewer considered the unequivocal or credible findings, which they grouped into categories based on their resemblance in meaning and concepts. Then, the categories with commonality were aggregated into synthesized categories by the primary reviewer and discussed by the second reviewer. Based on the categories, themes were developed inductively into synthesized categories by the primary reviewer and discussed by the second reviewer. The final synthesized findings illustrate the changes that related the findings to the physical, social, and mental domains of HRQL. The review team discussed and confirmed the final synthesized findings.

### Assessing confidence

Assessing confidence was based on the ConQual approach [30], as presented in Table 1. Five questions (Q2, Q3, Q4, Q6, and Q7) of the JBI SUMARI critical appraisal were considered to assess the dependability of the extracted findings. The level of dependability ranged from high (ten) to moderate (seven). In this review, eight studies [32–39] had a high level of dependability, meeting four or more of the criteria. Whereas five studies [40–44] had a medium level, satisfying two or more of the criteria. The credibility of the findings was obtained through the congruency between the author’s interpretation and supporting data. In this review, there was a combination of unequivocal and credible levels of evidence. Therefore, the findings were downgraded from high to moderate credibility.

**Table 1:** Dependability scores for included studies.

Dependability score						
Citation	Is there congruity between the research methodology and the research question or objectives	Is there congruity between the research methodology and the methods used to collect data?	Is there congruity between the research methodology and the representation and analysis of data?	Is there a statement locating the researcher culturally or theoretically?	Is the researcher’s influence on the research, and vice-versa, addressed?	Dependability score
Clark et al. (2005)	Yes	Yes	Yes	No	Yes	4/5 High
Dechaine et al. (2018)	Yes	Yes	Yes	Yes	Yes	5/5 High
Jokar et al. (2017)	Yes	Yes	Yes	No	No	3/5 Mod
Mead et al. (2010)	Yes	Yes	Yes	Yes	Yes	5/5 High
McPhillips et al. (2021)	Yes	Yes	Yes	Unclear	Unclear	3/5 Mod
Meredith et al. (2019)	Yes	Yes	Yes	Yes	Yes	5/5 High
Mitchel et al. (1999)	Yes	Yes	Yes	Unclear	Unclear	3/5 Mod
Nadarajah et al. (2017)	Yes	Yes	Yes	Yes	Yes	5/5 High
Nicolai et al., 2018	Yes	Yes	Yes	Unclear	Unclear	3/5 Mod
Pietrabissa et al. (2015)	Yes	Yes	Yes	No	No	3/5 Mod
White et al. (2010)	Yes	Yes	Yes	Unclear	Yes	4/5 Mod
White et al. (2011)	Yes	Yes	Yes	Unclear	Yes	4/5 Mod
Wong et al. (2016)	Yes	Yes	Yes	Yes	Yes	5/5 High

## Results

### Study inclusion

**Fig 1:**
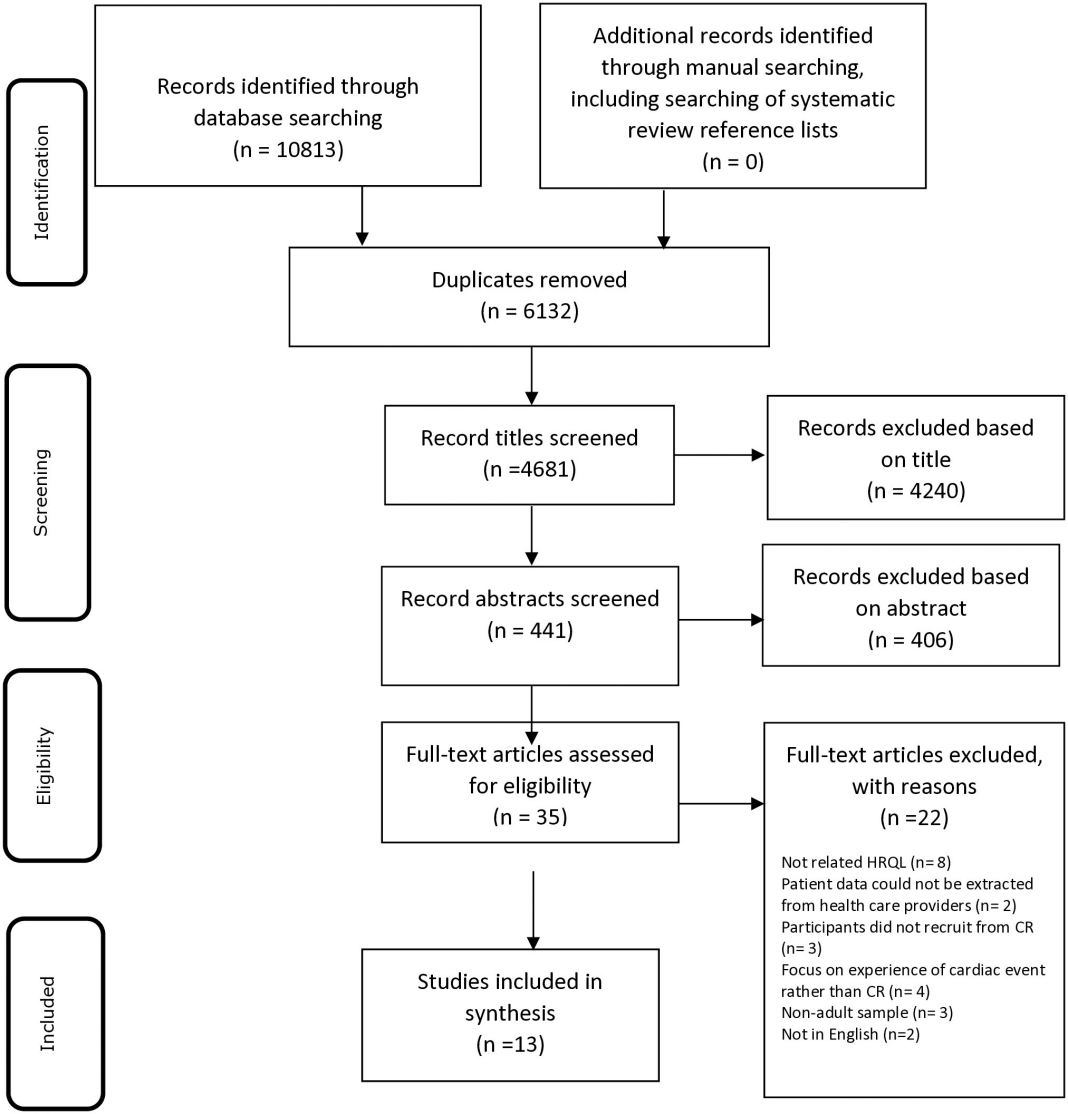
Study selection and PRISMA flow diagram [45].

A PRISMA flow diagram illustrating the selection of eligible studies is presented in Fig 1 (also see S3 File). A total of 10,813 articles were retrieved from a systematic search. Duplicates (n = 6132) were removed, so 4681 articles remained (See S3 File). Two independent (AR, LM) reviewers screened titles, after these two independent reviewers assessed 441 abstracts for suitability. Then, the full-text screening of 35 eligible articles was carried out for which members of the research team (LN, IJ, LW, HH, AR) each reviewed a sample of articles and assessed eligibility against criteria. This process was quality assured by a 2^nd^ reviewer (HH) who checked agreement with inclusion or exclusion of the studies. Finally, the lead author (AR) checked and confirmed the final articles to be included in the synthesis, which yielded 13 articles. (See S4 File Article Screening).

### Methodological quality of included studies

Of the 13 qualitative studies (maximum quality score 10), five studies were assigned a score of 7 [41–45], three studies scored nine [37, 39, 40], and the remaining studies scored ten [32–35, 37]. The philosophical perspective underlying the research design was explained in seven studies [32–36, 38, 39, 42]. The congruity between the research methodology and the research question was clearly stated in all included studies, as well as suitable methods for collecting and analyzing data. The cultural or theoretical perspective was highlighted in five studies [32–35, 37]. Only eight studies [32–39] identified the researcher’s influence on the research and vice-versa. A summary of the methodological quality of the studies is displayed in Table 2.

**Table 2:** Methodological quality of studies (S5 File).

Citation	Q1	Q2	Q3	Q4	Q5	Q6	Q7	Q8	Q9	Q10	Score /10
Clark et al. (2005)	Y	Y	Y	Y	Y	N	Y	Y	Y	Y	9/10
Dechaine et al. (2018)	Y	Y	Y	Y	Y	Y	Y	Y	Y	Y	10/10
Jokar et al. (2017)	N	Y	Y	Y	Y	N	N	Y	Y	Y	7/10
Mead et al. (2010)	Y	Y	Y	Y	Y	Y	Y	Y	Y	Y	10/10
McPhillips et al. (2021)	U	Y	Y	Y	Y	U	U	Y	Y	Y	7/10
Meredith et al. (2019)	Y	Y	Y	Y	Y	Y	Y	Y	Y	Y	10/10
Mitchel et al. (1999)	Y	Y	Y	Y	Y	U	U	Y	N	Y	7/10
Nadarajah et al. (2017)	U	Y	Y	Y	Y	Y	Y	Y	Y	Y	10/10
Nicolai et al. (2018)	U	Y	Y	Y	Y	U	U	Y	Y	Y	7/10
Pietrabissa et al. (2015)	N	Y	Y	Y	Y	N	N	Y	Y	Y	7/10
White et al. (2010)	Y	Y	Y	Y	Y	U	Y	Y	Y	Y	9/10
White et al. (2011)	Y	Y	Y	Y	Y	U	Y	Y	Y	Y	9/10
Wong et al. (2016)	Y	Y	Y	Y	Y	Y	Y	Y	Y	Y	10/10

Key: Y, yes; N, no; U, unclear.

Questions:

1. Is there congruity between the stated philosophical perspective and the research methodology?

2. Is there congruity between the research methodology and the research question or objectives?

3. Is there congruity between the research methodology and the methods used to collect data?

4. Is there congruity between the research methodology and the representation and analysis of the data?

5. Is there congruity between the research methodology and the interpretation of the results?

6. Is there a statement locating the researcher culturally or theoretically?

7. Is the influence of the researcher on the research, and vice-versa, addressed?

8. Are participants and their voices adequately represented?

9. Is the research ethical, according to current criteria, or for recent studies, and is there evidence of ethical approval by an appropriate body?

10. Do the conclusions drawn in the research report flow from the analysis or interpretation of the data?

### Characteristics of included studies

Five studies were conducted in England [32, 34, 38, 39, 41], three studies in the United States of America [33, 37, 42] and one study each in Scotland [36], Iran [40], Italy [44] Germany [43] and Hong Kong [35]. Nine studies collected data using interviews [32, 34, 35, 37–44], two studies used focus groups [33, 36], and one study used three methods to collect data, including participant observation, an interview, and a reflexive diary [34]. For the list of study findings relevant to more than 12 weeks of CR, See S6 File, for the Characteristics of included studies for methodological review

### Review findings

Synthesized findings consisted of 3 categories generated from 39 findings from 13 papers (See S7 File for Data Extracted and File 8 for the summary of themes). Three main findings were identified.

### Building healthier habits

This category contained findings extracted from 8 studies [35–40, 42, 43]. CR was described as a platform for changing lifestyles and an experience that allowed participants to increase their knowledge and greater understanding of their physical strengths and limitations. A participant outlined the consequences of participating in CR: “My *whole pattern of life has changed*. *I had never been to a gym before*, *and suddenly I have found I am enjoying it and of course*, *it is geared for my age group and my particular problem*. *Before*, *you didn’t know your limitations*, *but after*, *definitely after doing rehabilitation*, *you could tell exactly what you could do*. *I now know there is lots I can do*” [36, 366]. Experiencing health benefits enhanced participants’ views on their ability to exercise: *“Otherwise*, *I wouldn’t ride my bicycle or quit smoking*. *To be honest*, *if I didn’t gain something positive*, *I wouldn’t do it*” [43, 31]. Two studies [37, 39] reported that CR programs were effective in reducing fat, losing weight, or eating less, leading to maintaining a healthy lifestyle. One participant stated: *“I’m cutting things out*, *puddings and sugary things*, *fatty things*, *so I’m being more careful with my diet*” [39,124]. Another participant commented that “*because I went on this diet*, *I have an excuse to say to some of my friends [at a previous job]*, *‘I don’t want to go to lunch every week*.*’ I go to exercise Monday*, *Wednesday*, *Friday*, *and I am in here [CR] from 11 am to 1 pm*” [37, 236]. CR programs were perceived as positive and encouraged healthy behavior to change behavioral habits and resume a normal life: “*I liked CR*. *It built up my exercise habit*, *and I lost some weight*, *felt stronger in my leg muscles*, *and trained my arm*. *Exercise is good for my health*, *especially for my heart … I think it can help me to resume my health*” [35, 729]. The direct benefit of engaging in exercise was associated with a greater sense of well-being: "*I feel better now than I ever did before*, *now my heart is normal after four years of exercise*” [42, 238]. CR encouraged patients to implement exercise in their daily lives. Therefore, the transition from CR to daily life implies continuity of maintaining a healthy lifestyle: “I’ve followed an exercise regime since I left the rehab programme, *and I do cardiovascular sort of running exercises and walking*” [38, 51]. Patients also believed that continuing the CR program helps them maintain their health: “I aim *to reach that number before my final session*. *Next*, *I would like to try my best to maintain this positive outcome even after I leave here*. *I would also like to continue doing vigorous exercises in order to maintain my health”* [40, 348]. CR was identified as a space that enhanced patients’ good health and improved their vitality and energy*: “The rehab has been making me feel better. I’m tired in the morning, but when I leave, I’m full of energy” [33, 72].*

### Increasing levels of peer interaction

Four studies discussed the benefits of peer interaction at CR [34–36, 42]. CR created a forum for peer interaction. Participants enjoyed regular contact with and accompanying other users while attending CR: “*I enjoyed coming to the class and meeting people*. *I would have liked it to go on longer socially*. *Once you finish the class*, *miss coming to the classes and the accompany more than anything*” [36, 367]. Patients valued sharing the experience with other members. It was beneficial for them to connect with other patients who amplified their emotions, making their positive experiences more positive and increasing their enjoyment. For example: “*I was getting better*, *and I wanted something to show me that I would have a ‘normal’ life Rehab has really helped with this*, *and the other people that are in here*. *It’s just nice to have a group of people with a shared experience that can have a laugh and a joke and have a chat about the issues*” [34, 371]. Patients appreciated the opportunity to share both challenges and successes with helpful staff. This network appeared to mitigate loneliness and hopelessness: “*Sometimes*, *you just need someone to listen to you*. *If you feel like crying*, *just cry*. *You think you are in that boat by yourself*, *but all of us are riding in that same boat”* [33, 79]. Social contact with other members of the CR programme appeared to be important for patients in maintaining exercise behavior. Undertaking exercise with peer and group dynamics strengthened the positive belief in the value of engagement in exercise long-term: “*I’ve made a lot of friends here*, *and that’s what keeps me coming back*” [42, 238]. Patients commented on how CR provided a space where they could exercise in a group with peers who also have the potential to help them maintain physical activity after CR: *“Exercise peers in CR are important for me to keep joining until completion of the CR class*. *Now we always meet and join some other low-level hiking activities together … Walking together is fun*, *and we can talk about everything when we walk”* [35, 729].

### Improving mental health

Findings from five studies [33, 34, 36, 40, 41, 44] informed this category. CR programmes were described as fostering a sense of meaning in life, which, in turn, improved mental health. Patients perceived that CR programs provided compassionate care, fostered positive emotions, and encouraged a positive mindset: “You *know*, *I didn’t realize how miserable I was before coming here*. *I had to try very hard not to bite someone’s head off; my temper became short*. *But I didn’t give up; I think anybody else would have given up a very long time ago*. *I keep wrestling with my mind so that it doesn’t get me down*. *I am getting to know myself better”* [34]. CR programmes help patients to change their thoughts, reduce worry and enhance positive thinking: “*It teaches you how to not have to worry*, *or worry as much*, *or feel anxious and scared*, *how to postpone those thoughts… how to push ‘em’ away and try to get to a certain percentage of positive thinking*” [41, 5]. Personal confidence and fitness level increased as a result of attending CR: “*you would be talking to the other people (at CR) and we’re all in the same boat*. *I felt it built up your confidence and get fitter*” [36, 367]. The CR environment fostered hope for a longer life and the ability to achieve goals: “*I am feeling much better*. *Now*, *I have many things to live for*. *I want to see my daughter getting married; I’d like to live long enough to see my grandkids*. *We all need is something to push us forward*, *someone to exercise for*, *to live for*. *I will do my best to listen to any advice that helps me rehabilitate for the sake of not only my own health but also of my loved ones* [40, 349]. Some perceived CR as the vehicle to living well for longer and maintaining personal and family connections: “*I’m looking at it as longevity; I don’t want to sit in a wheelchair somewhere where I’m incontinent*, *and I’m contributing nothing*. *I want a future*. *… I want to be there with my husband when he finally retires* [32, 496], and patients commented on how CR helped them to return to a healthy life *“to get back to a healthier status and spend more time with my children*…*with my wife too*!” [44, 4].

## Discussion

The purpose of this review was to explore the perceptions of patients with heart disease about the changes in their HRQL following attendance at CR. Thirteen studies were included in this review that met the inclusion criteria, and these studies were reported only from patients’ perspectives. Therefore, this review described the patients’ perspectives on positive changes after attending CR. The changes were associated with building healthier habits, increasing peer interaction, and improving mental health. To illustrate the changes, we related the findings to the physical, social, and cognitive domains of HRQL.

CR was described as one of the key elements for building healthier habits, which positively impacted the patient’s lives, primarily in the physical domain. Restarting a regular exercise routine, eating a nutritious diet, or complying with dietary restrictions were the key features that patients described as beneficial. These benefits aligned with feeling better in oneself and improving well-being, which helped people improve their physical health through their behavior and maintain healthy habits. Further studies have indicated that adopting a healthy lifestyle improved the functional capacity and HRQL of patients with heart disease [11, 46]. However, the findings also highlight the value of health professionals, especially nurses, supporting patients’ motivation to maintain healthier habits post-CR by highlighting the improvement in physiological measures, for example, weight and fitness.

Peer interactions during CR can substantially promote mental and physical domains of HRQL among patients with heart disease. In the CR setting, therapeutic alliances were developed and sustained. An environment that encouraged peer interaction between the group members to share their positive and negative experiences of making lifestyle changes was created, and some maintained connections beyond the CR program. It is also suggested that families can be considered peers who can optimize the rehabilitation journey by promoting an active and healthy lifestyle and encouraging engagement in physical activity across South Asian and Caucasian communities [47–49]. During CR, patients had an opportunity to enhance their peer networks. Peer networks provided emotional support to other participants, which helped motivate them to make lifestyle changes and maintain healthy behavior. Parallel to this review, the study by Anttila et al. [50] indicated that socialization and bonding with other patients was an important component of the CR program and contributed to the favorable effects on the psychological well-being of patients with heart disease [50]. Also, peer interaction between the group members in the CR helped them to understand the rationale for changes better and obtain the skills to maintain healthy behaviors in the long term. Interacting with other members was described as a positive factor, providing an environment where people could meet other members similar to themselves at CR. This is also in line with a study that found peers have good potential to help patients with heart disease sustain physical activity levels with greater cardiovascular benefit after CR [51]. Sharing experiences influenced how patients communicate with others through increased displays of emotion, which allowed them to improve social communication and express their feelings. This sharing was considered part of the curing process that could assist patients in normalizing feelings. However, the lack of recognition and integration of peer support in CR was identified by the recent study [52], highlighting the importance need of peer support during the rehabilitation process, which can lead to better rehabilitation outcomes for older patients [52].

Another positive change was related to the patient’s mental health. This change was commonly conceptualized as hope and confidence, which acted as potential mechanisms for achieving positive mental health. Prior studies have shown that CR provided an opportunity to help patients improve their mental health care and even obtain treatment for mental disorders [53]. Likewise, another study reported an improvement in mental quality of life and lower depression among patients with heart disease [54]. A recent Australian study indicated that CR program could reduce stress and depression by addressing emotional well-being and promoting motivation, and ultimately enhance adherence to attending CR [55]. Besides this, a recent quantitative study highlighted that CR plays a significant role in improving HRQL as part of assessing patient-reported outcome measures to support mental health and helps to ensure the maintenance of higher level of mental health quality [56].

CR provided a recovery space for patients to foster the hope of prolonging their lives and continue living longer. This review indicated that patients were more confident managing their health after attending the CR programme. This was reflected by regaining their independence and thus encouraging them to keep a healthy lifestyle. In addition, in CR, negative thoughts substituted positive thoughts and prevented situations that triggered unpleasant thoughts and emotions, contributing to positive behavior changes. CR led to clinically significant improvement in quality of life and physical and mental activity status [57].

### Strengths and limitations

This review implemented a comprehensive systematic search across numerous databases, and the lack of date restrictions ensured a comprehensive inclusion of studies, maximizing the scope of available evidence. We followed PRISMA guidelines, ensuring transparency and methodological rigor throughout the review process. The synthesized findings were divided into distinct categories, highlighting key areas of improvement in patients’ health-related quality of life after cardiac rehabilitation, including mental and physical aspects. The authors of this review are from Australia (AR, LW, HH, LM) and the UK (IJ, LN), and include health professionals and academic researchers from diverse fields. Their expertise spans cardiovascular nursing (AR, LW, HH, IJ), health psychology (LN), and library services (LM). This multidisciplinary team brought diverse perspectives to interpreting the findings, enriching the overall analysis. The review highlighted the role of peer interaction, an often-under-researched area, as a key factor in improving mental and physical health. However, this review has some limitations that should be noted. First, by excluding non-English studies, the experiences of individuals in non-English speaking countries may not be represented. Second, while the included studies addressed changes in HRQL following CR, variations in study methodologies and data collection procedures may have made effective data synthesis challenging. Additionally, the exclusion of quantitative studies and the homogeneity of study characteristics, target populations, and CR settings may limit the broader applicability of the findings.

### Implications and future research

Given the importance of peer support in CR, it is recommended that greater emphasis be placed on promoting and integrating peer support into CR programs to improve health-related quality of life (HRQL) and optimize patient outcomes. Clinicians and policymakers should recognize the unique value of peer support and explore which aspects are most beneficial in supporting patients. Stakeholders should aim to offer diverse forms of peer support and facilitate its widespread adoption and scaling. Additionally, more targeted mental health programs should focus on individuals in need, with healthcare professionals dedicating more time and resources to these programs to strengthen positive thinking, foster independence, and support maintaining a healthy lifestyle. Further research is needed to understand how to empower patients to commence and complete CR programs and to capture changes in HRQL at multiple consecutive. It would be beneficial for future research to explore how patients can be supported to maintain their increased HRQL and sustain their behavior change long-term, post-CR. Furthermore, follow-up studies at long-term intervals could examine the sustained effect of CR. Involving family members as critical partners in the caring plan could also add a broader measurement of HRQL.

## Conclusion

This review described positive changes in HRQL due to undertaking CR programs among patients with heart disease. These changes were associated with the social, physical, and mental health of HRQL of patients with heart disease. Based on the discussion of the findings, peer interaction with other CR members improved both the psychological and physical health of heart disease patients. Also, this review indicated that adopting healthier habits, including healthy eating and regular physical activity, had substantial benefits in formulating healthy behavior. Adaptation and maintenance of healthy habits over the long term reinforced the implementation of behavioral change. Given the prominent role of peers in contributing to a healthy lifestyle, including peers in lifestyle advice appears to be an avenue towards improving mental health. The findings of this review provide new and important knowledge on the benefits of participation in the CR from patients’ perspectives. Nurses and other health professionals are critical in encouraging patients to attend CR. Awareness of patient’s perceptions is essential to assist patients to make and maintain healthy habits effectively. The study also demonstrates new insight into regaining confidence in patients and helps in finding ways back to a new normality.

## Supporting information

S1 File Prisma checklist [58]. (DOC)

S2 File Search strategy [58]. (DOC)

S3 File Screened articles [58]. (XLS)

S4 File Article screening [58]. (DOC)

S5 File Dependability assessment of included studies [58]. (DOC)

S6 File Characteristics of included studies for methodological review [58]. (DOC)

S7 File Data extraction; and file [58]. (DOC)

S8 File Themes and categories [58]. (DOC)
