# Supplementary material for: The changes in health-related quality of life after attending cardiac rehabilitation: A qualitative systematic review of the perspective of patients living with heart disease
Source: PLoS One. 2025 Jan 30;20(1):e0313612. doi: 10.1371/journal.pone.0313612 (PMC11781667; doi:10.1371/journal.pone.0313612)
Supplement: S2 File — (DOC) [file pone.0313612.s002.DOC]

**Supplementary File 2: search strategy**

**CINAHL**

(("heart disease*") OR ("heart failure*") OR ("coronary disease*") OR ("coronary artery disease*") OR ("cardiovascular disease*") OR ("myocardial Ischemia*") OR angina* OR ((MH "Cardiovascular Diseases")) OR ((MH "Heart Diseases")) OR ((MH "Myocardial Ischemia+")) OR ((MH "Coronary Disease+")) OR ((MH "Myocardial Diseases+")) OR ((MH "Heart Failure+")) OR ((MH "Cardiac Patients")) OR ((MH "Heart Surgery+"))) AND ((health n6 improve*) OR ("overall health") OR (wellbeing OR "well being") OR ("life quality") OR ("health related quality of life" OR HRQL) OR ("quality of life" OR QoL) OR ((MH "Quality of Life")) OR ((MH "Outcomes (Health Care)")) OR ((MH "Patient-Reported Outcomes")) OR ((MH "Self Report")) OR ("self report")) AND (("cardiac rehabilitation") OR ("cardiac* exercise") OR ("cardiac intervention*") OR ("secondary prevention") OR ((MH "Preventive Health Care")) OR ((MH "Rehabilitation, Cardiac+")) OR ((MH "American Association of Cardiovascular and Pulmonary Rehabilitation")) OR ((MH "Therapeutic Exercise")))

4,250

**Medline**

(("heart disease*") OR ("heart failure*") OR ("coronary disease*") OR ("coronary artery disease*") OR ("cardiovascular disease*") OR ("myocardial ischemia*") OR angina OR ((MH "Cardiovascular Diseases")) OR ((MH "Heart Diseases")) OR ((MH "Myocardial Ischemia+")) OR ((MH "Coronary Disease+")) OR ((MH "Cardiomyopathies+")) OR ((MH "Heart Failure+")) OR ((MH "Thoracic Surgery"))) AND (("quality of life" OR QoL) OR (health n6 improve*) OR ("overall health") OR (wellbeing OR "well being") OR ("life quality") OR ("health related quality of life" OR HRQL) OR ("self report") OR ((MH "Quality of Life")) OR ((MH "Outcome Assessment, Health Care")) OR ((MH "Patient Reported Outcome Measures")) OR ((MH "Self Report"))) AND (("cardiac rehabilitation") OR ("cardiac* exercise") OR ("cardiac intervention*") OR ("secondary prevention") OR ((MH "Secondary Prevention")) OR ((MH "Cardiac Rehabilitation")) OR ((MH "Exercise Therapy")))

**PsycINFO**

(("heart disease*") OR ("heart failure*") OR ("coronary disease*") OR ("cardiovascular disease*") OR ("coronary artery disease*") OR ("myocardial ischemia*") OR angina* OR (DE "Cardiovascular Disorders") OR (DE "Heart Disorders" OR DE "Angina Pectoris" OR DE "Arrhythmias (Heart)" OR DE "Coronary Thromboses" OR DE "Myocardial Infarctions") OR (DE "Heart Surgery")) AND (("quality of life" OR QoL) OR (health n6 improve*) OR ("overall health") OR (wellbeing OR "well being") OR ("life quality") OR ("health related quality of life" OR HRQL) OR ("self report*") OR (DE "Quality of Life" OR DE "Health Related Quality of Life") OR (DE "Treatment Process and Outcome Measures") OR (DE "Patient Reported Outcome Measures") OR (DE "Well Being") OR (DE "Self-Report")) AND (("cardiac rehabilitation") OR ("cardiac* exercise*") OR ("cardiac intervention*") OR ("secondary prevention") OR (DE "Rehabilitation") OR (DE "Exercise"))

550

**Embase**

Query ('heart disease'/de OR 'heart disease' OR 'heart failure'/exp OR 'heart failure' OR 'coronary disease' OR 'coronary artery disease'/exp OR 'coronary artery disease' OR 'cardiovascular disease'/de OR 'cardiovascular disease' OR 'heart muscle ischemia'/exp OR 'heart muscle ischemia' OR 'myocardial ischemia' OR 'angina pectoris'/de OR 'angina pectoris' OR 'angina' OR 'myocardial disease'/exp OR 'myocardial disease' OR 'cardiac patients'/de OR 'cardiac patients' OR 'cardiovascular surgery'/exp OR 'cardiovascular surgery') AND ('quality of life'/exp OR 'quality of life' OR (health NEAR/6 improve*) OR 'overall health' OR 'wellbeing'/de OR 'wellbeing' OR 'well being' OR 'life quality' OR 'health related quality of life' OR hrql OR 'self report'/de OR 'self report' OR 'patient-reported outcome'/de OR 'patient-reported outcome' OR 'outcome assessment'/exp OR 'outcome assessment') AND ('cardiac rehabilitation' OR 'cardiac exercise' OR 'cardiac intervention' OR 'secondary prevention' OR 'heart rehabilitation'/de OR 'heart rehabilitation')

**JBI**

1 (heart disease* or cardiovascular or coronary or myocardial or cardiac).mp. [mp=text, heading word, subject area node word, title]

2 Life quality.mp. [mp=text, heading word, subject area node word, title]

3 health related quality of life.mp. [mp=text, heading word, subject area node word, title]

4 (wellbeing or well being).mp. [mp=text, heading word, subject area node word, title]

5 exercise.mp. [mp=text, heading word, subject area node word, title]

6 intervention.mp. [mp=text, heading word, subject area node word, title]

7 cardiac rehabilitation.mp. [mp=text, heading word, subject area node word, title]

8 secondary prevention.mp. [mp=text, heading word, subject area node word, title]

9 2 or 3 or 4

10 5 or 6 or 7 or 8

11 1 and 9 and 10

**Cochrane Library**

("heart disease*" OR "heart failure*" OR "cardiovascular disease*" OR "myocardial ischemia" OR "cardiovascular patient*" OR "cardiovascular surgery*")

AND ("health related quality of life" OR "quality of life" OR wellbeing OR "well being" OR "life quality" OR "overall health" OR "health outcome*" OR "patient report*")

AND ("cardiac rehabilitation" OR "cardiac exercise" OR "secondary prevention" OR "cardiac intervention*" OR "prevent* health" OR "therap* exercise")

**SCOPUS**

(TITLE-ABS-KEY("heart disease*" OR "heart failure*" OR "cardiovascular disease*" OR "myocardial ischemia" OR "cardiovascular patient*" OR "cardiovascular surgery*")

AND TITLE-ABS-KEY("health related quality of life" OR "quality of life" OR wellbeing OR "well being" OR "life quality" OR "overall health" OR "health outcome*" OR "patient report*")

AND TITLE-ABS-KEY("cardiac rehabilitation" OR "cardiac exercise" OR "secondary prevention" OR "cardiac intervention*" OR "prevent* health" OR "therap* exercise"))

**3,608**

**Web of Science**

("heart disease*" OR "heart failure*" OR "cardiovascular disease*" OR "myocardial ischemia" OR "cardiovascular patient*" OR "cardiovascular surgery*")

AND ("health related quality of life" OR "quality of life" OR wellbeing OR "well being" OR "life quality" OR "overall health" OR "health outcome*" OR "patient report*")

AND ("cardiac rehabilitation" OR "cardiac exercise" OR "secondary prevention" OR "cardiac intervention*" OR "prevent* health" OR "therap* exercise")
