# Supplementary material for: The changes in health-related quality of life after attending cardiac rehabilitation: A qualitative systematic review of the perspective of patients living with heart disease
Source: PLoS One. 2025 Jan 30;20(1):e0313612. doi: 10.1371/journal.pone.0313612 (PMC11781667; doi:10.1371/journal.pone.0313612)
Supplement: S4 File — (DOC) [file pone.0313612.s004.DOC]

**Supplementary File 4: Full text Article Screening**

| **ID** | **Author** |  | **Full text screening** | | | | |
| --- | --- | --- | --- | --- | --- | --- | --- |
|  |  | **Title** | **Include?** | **Exclude?** | **Reason for exclusion** |  |  |
| L.W | Alvai | Barriers to education in cardiac rehabilitation within an Iranian society: A qualitative descriptive study |  | Yes | Does not include any data relating to HRQl . Focus is on delievry CR | | |
|  | Adams | Importance of resistance training for patients after a cardiac event |  | Yes | Wrong outcome | | |
| **L.W** | **Clark** | **A realist study of the mechanisms of cardiac rehabilitation** | **Yes** |  |  |  |  |
|  | Boothby | Sexual Activity After Acute Coronary Syndrome: A Qualitative Approach to Patient and Partner Experiences |  | Yes | Wrong outcome | | |
|  | Balestroni | Lifestyle and adherence to the recommended treatments after cardiac transplantation |  |  | Wrong outcome | | |
|  | Devi | Exploring the experience of using a web-based cardiac rehabilitation programme in a primary care angina population: a qualitative study |  | Yes | Qualitative, psychological wellbeing not QoL. For discussion. | | |
|  | Grace | Patient preferences for home-based versus hospital-based cardiac rehabilitation |  | Yes | Quant study and focus more on delivering CR | | |
| **L.W** | **Dechaine.** | **Healing the heart: A qualitative study of challenges and motivations to cardiac rehabilitation attendance and completion among women and men** | **Yes** |  |  |  |  |
|  | Gulanick | Recovery patterns and lifestyle changes after coronary angioplasty: the patient's perspective |  |  | Not focus on HRLQ | | |
|  | Hummel | Quality of life after heart and heart-lung transplantation |  | Yes | wrong outcome | | |
| L.W | Helem | “When what is taken for granted disappears”: women’s experiences and perceptions after a cardiac event |  | Yes | wrong outcome | | |
| L.W | Jbilo | Understanding men's psychological reactions and experience following a cardiac event: A qualitative study from the MindTheHeart project |  | Yes | Not focus on HRLQ | | |
| L.W | Jokar | Behavioral change challenges in the context of center-based cardiac rehabilitation: A qualitative study |  | Yes |  |  |  |
| **L.W** | **Jokar** | **Begin again and continue with life: A qualitative study on the experiences of cardiac rehabilitation patients** | **Yes** |  |  |  |  |
| L.N | King | Men and women managing coronary artery disease risk: Urban-rural contrasts |  | Yes | wrong outcome | | |
| L.N | Leung | Gender differences in motivations and perceived effects of Mind-Body Therapy (MBT) practice and views on integrative cardiac rehabilitation. |  | Yes | wrong outcome | | |
| **L.N** | **Mcphilips** | **Cardiac rehabilitation patients experiences and understanding of group metacognitive therapy: a qualitative study** | **Yes** |  |  |  |  |
| **L.N** | **Mead** | **Barriers to effective self-management in cardiac patients: The patient's experience** | **Yes** |  |  |  |  |
|  | Morton | Multidisciplinary team approach to heart failure management |  | Yes | Wrong outcome | | |
| **L.N** | **Meredith** | **Getting to the heart of the matter: an ethnography of emotions and emotion regulation in cardiac rehabilitation** | **Yes** |  |  |  |  |
| **L.N** | **Mitchel** | **Cardiac rehabilitation: participating in an exercise program in a quest to survive** | **Yes** |  |  |  |  |
| **I.J** | **Nadarajah** | **The lived experience of individuals in cardiac rehabilitation who have a positive outlook on their cardiac recovery** | **Yes** |  |  |  |  |
| **L.N** | **Nicolai** | **To change or not to change - That is the question: A qualitative study of lifestyle changes following acute myocardial infarction** | **Yes** |  |  |  |  |
| I.J | Ohagan | Work Reintegration and Cardiovascular Disease: Medical and Rehabilitation Influences |  | Yes | Wrong outcome | | |
|  | **Parkosewich** | **Cardiac rehabilitation barriers and opportunities among women with cardiovascular disease** |  | Yes | Wrong outcome | | |
| I.J | Paquet | Re-engineering cardiac rehabilitation programmes: Considering the patient's point of view |  | Yes | Wrong outcome | | |
| **I.J** | **Pietrabissa.** | **Enhancing behavioral change with motivational interviewing: A case study in a Cardiac rehabilitation unit** | **Yes** |  |  |  |  |
|  | Rushford | Recall of information received in hospital by female cardiac patients |  | Yes | Wrong outcome | | |
| I.J | pulignano | Barriers to Cardiac Rehabilitation access of older heart failure patients and strategies for better implementation |  | Yes | Wrong outcome | | |
|  | Smirnov | Effect of occupational therapy on the psychosocial status of patients with myocardial infarction at the second stage of rehabilitation |  | Yes | Wrong outcome | | |
|  | Tolmie | Coronary artery bypass graft operation: patients' experience of health and well-being over time |  | Yes | Health and well being not QoL specifically | | |
| I.J | Smith | ‘Betwixt and between health and illness’ – women's narratives following acute coronary syndrome |  | Yes |  |  |  |
|  | **White** | **Patients' perspectives on cardiac rehabilitation, lifestyle change and taking medicines: Implications for service development** | **Yes** |  |  |  |  |
|  | **White** | **Qualitative study of cardiac rehabilitation patients' perspectives on making dietary changes.** | **Yes** |  |  |  |  |
|  | **Wong** | **Attitude toward the out-patient cardiac rehabilitation program and facilitators for maintenance of exercise behavior** | **Yes** |  |  |  |  |
|  | Yu | Long-term changes in exercise capacity, quality of life, body anthropometry, and lipid profiles after a cardiac rehabilitation program in obese patients with coronary heart disease |  | Yes | Quan study and focus more on delivering CR | | |
|  |  |  |  |  |  |  |  |
