# Supplementary material for: The changes in health-related quality of life after attending cardiac rehabilitation: A qualitative systematic review of the perspective of patients living with heart disease
Source: PLoS One. 2025 Jan 30;20(1):e0313612. doi: 10.1371/journal.pone.0313612 (PMC11781667; doi:10.1371/journal.pone.0313612)
Supplement: S5 File — (DOC) [file pone.0313612.s005.DOC]

**Supplementary File 5: Dependability Assessment of included studies**

| Dependability score | | | | | | |
| --- | --- | --- | --- | --- | --- | --- |
| Citation | Is there congruity between the research methodology and the research question or objectives | Is there congruity between the research methodology  and the methods used to collect data? | Is there congruity between the research methodology and the representation and analysis of data? | Is there a statement locating the researcher culturally or theoretically? | Is the influence of the researcher on the research, and vice-versa, addressed? | Dependability score |
| Clark *et al.* (2005) | Yes | Yes | Yes | No | Yes | 4/5 High |
| Dechaine *et al.* (2018) | Yes | Yes | Yes | Yes | Yes | 5/5 High |
| Joker *et al.* (2017) | Yes | Yes | Yes | No | No | 3/5 Mod |
| Mead et al. (2010) | Yes | Yes | Yes | Yes | Yes | 5/5 High |
| McPhillips et al. (2021) | Yes | Yes | Yes | Unclear | Unclear | 3/5 Mod |
| Meredith *et al.* (2019) | Yes | Yes | Yes | Yes | Yes | 5/5 High |
| Mitchel *et al.* (1999) | Yes | Yes | Yes | Unclear | Unclear | 3/5 Mod |
| Nadarajah *et al.* (2017) | Yes | Yes | Yes | Yes | Yes | 5/5 High |
| Nicolai et al., 2018 | Yes | Yes | Yes | Unclear | Unclear | 3/5 Mod |
| Pietrabissa et al,(2015) |  |  |  |  |  |  |
| White et al (2010) | Yes | Yes | Yes | Unclear | Yes | 4/5 Mod |
| White et al (2011) | Yes | Yes | Yes | Unclear | Yes | 4/5 Mod |
| Wong *et al.(2016)* | Yes | Yes | Yes | Yes | Yes | 5/5 High |
