# Supplementary material for: The changes in health-related quality of life after attending cardiac rehabilitation: A qualitative systematic review of the perspective of patients living with heart disease
Source: PLoS One. 2025 Jan 30;20(1):e0313612. doi: 10.1371/journal.pone.0313612 (PMC11781667; doi:10.1371/journal.pone.0313612)
Supplement: S6 File — (DOC) [file pone.0313612.s006.DOC]

**Supplementary File 6: Characteristics of included studies for methodological review**

| **Study** | **Methods for data collection and analysis** | **Country** | **Phenomena of interest** | **Setting / context** | **Participant characteristics and sample size** | **Description of main results** |
| --- | --- | --- | --- | --- | --- | --- |
| Clark *et al.* (2005) | Focus groups were conducted to discuss their perceptions and experiences. The data were generated and analysed using the  realist approach of Pawson and Tilley. | Scotland | To explore patients’ experiences of cardiac rehabilitation  and perceptions of the mechanisms and contexts influencing its long-term effectiveness. | Cardiac rehabilitation | Forty-seven participants (30 males and 17 females) with a formal diagnosis of coronary heart disease | Results. Participants’ accounts indicated that the didactic content of cardiac reha-  bilitation was not strongly linked to longer-term health behaviour change. The main positive effects of cardiac rehabilitation were related to the effect of participation on mediating social and body-focused mechanisms that were triggered when the reha- bilitation setting was perceived to be safe. Social mechanisms identified included social comparisons, camaraderie, and social capital. Body-focused mechanisms included greater knowledge of personal physical boundaries and a greater trust in the heart- diseased body. Collectively, these mechanisms had a positive effect on confidence that  was perceived as being imperative to maintain health behaviour change. |
| Dechaine *et al.* (2018) | Participants were equally divided based on the number of CR session completed (less than or equal to 18 vs. more than 18). Data were gathered from individual interviews and analysed using an inductive content analysis. | United States | To articulate similarities and differences in the CR treatment experiences by gender. | Urban and rural Cardiac rehabilitation | 20 women and 20 men | Both women and men view CR as a means to move forward from their cardiac incident and  achieve good health. Women were more likely than were men to be motivated by a perceived obligation to CR staff and improved quality of life. Men, in comparison, identified their commitment to CR as a need to complete a task. |
| Joker *et al.* (2017) | descriptive qualitative approach, which  is widely acknowledged as the best approach for eliciting in-depth  descriptions of behavioural changes in the context of  cardiac rehabilitation centres. | Iran | To explore the mechanism that underline behavioural changes within CR centres. | Cardiac rehabilitation | 15  men and 8 women with coronary heart disease | Trying to stay alive and begin again arose as the  two main themes related to behavioural change. These  themes include the changes in cognition and values that  lead to improvements in the process of behavioural change as  a major outcome of cardiac rehabilitation. |
| Mead et al. (2010) | Using a  qualitative study design consisting of focus group interview | United States | To explore how for disadvantaged patients access barriers interfere with typical  management challenges to undermine patients’ efforts to care for their illnesses. | Cardiac rehabilitation | 33 focus group discussions with heart patients | We identiﬁed nine major themes reﬂecting issues related to patients’ ability to care for and  manage their heart conditions. We grouped the themes into three domains of interest: (1) barriers that  interfere with getting necessary services, (2) barriers that impede the monitoring and management of a  heart condition on a daily basis, and (3) supports that enable self-management and improve care.  Conclusion: For disadvantaged populations, typical problems associated with self-management of a  heart condition are aggravated by substantial obstacles to accessing care. |
| McPhillips et al. (2021) | Using qualitative study with intervention component and using interview. | England | To explore qualitatively, CR  patients’ experiences and understanding of group MCT with  the aim of examining aspects of treatment that patients  experienced as helpful | Cardiac rehabilitation | 43 women and  Men with differing levels of distress and were approached  by telephone  from both the control and intervention. | Results Two main themes were identified: (1) general  therapy factors that were seen largely as beneficial, where  patients highlighted interaction with other CR patients  and CR staff delivery of treatment and their knowledge of  cardiology; (2) group MCT- specific factors that were seen  as beneficial encompassed patients’ understanding of the  intervention and use of particular group MCT techniques.  Most patients viewed MCT in a manner consistent with the  metacognitive model. All the patients who completed group  MCT were positive about it and described self- perceived  changes in their thinking and well- being. A minority of  patients gave specific reasons for not finding the treatment  helpful. |
| Meredith *et al.* (2019) | Three main methods were used to  collect data over a 12-month period, including participant observation (225 h),  informal and formal interviews, and a reﬂexive diary. Thematic analysis was  used to generate patterns (themes) in the data and purposeful sampling was purposeful | England | To provide  a penetrative insight into the social and psychological environment in a UK CR setting, with  a speciﬁc focus on aﬀective and behavioural phenomena. | Cardiac rehabilitation | 10 formal interviews were conducted to further elucidate the lived experiences of  several key actors within the CR context., interviewing patients  (n = 4), staﬀ (n = 3) and volunteers (n = 3). | Composite  narratives illustrated the emotional intensity of recovering from a cardiac  event and highlighted the value of CR to aid patients with reskilling and  emotional support. In discussing our data, we emphasise the potential value  of emotional intelligent care provision, and the creation of an environment  that encourages emotional disclosure. |
| Mitchel *et al.* (1999) | Adhering to the belief that human experience can be explained  from the viewpoint of the person who has experienced the event, this study attempted to explore and describe  strategies used by individuals who had experienced a cardiac  event to overcome barriers associated with an exercise program. Each interview last 1 hour. | United States | To explore and describe strategies that individ­  uals who experienced a cardiac event used to overcome barri­  ers associated with an exercise program. | Cardiac rehabilitation | Six subjects (four females and two males) who were enrolled  in a cardiac exercise programfor1 year or longer, and who con tinued to actively participate in at least two cardiac exercise  classes per year | Individuals who consistently participated in a cardiac exercise program did not use  strategies to overcome barriers; rather, in their quest to survive,  they used strategies to ensure their participation in the program. |
| Nadarajah *et al.* (2017) | Husserlian phenomenology guided this study. A purposive sample of 10 individuals who had experienced an  acute cardiac event and had a predominantly positive outlook were interviewed. Data were analyzed using Colaizzi’s  method. A purposive sampling strategy was used to recruit participants for this  study. | United States | To explore the experiences of cardiac recovery in cardiac rehabilitation participants with a  predominantly positive outlook, within the context of an acute cardiac event, including exploring barriers and facilitators  of cardiac recovery.  And to explore how a positive outlook impacted completion of phase two of the  cardiac rehabilitation program | Cardiac rehabilitation | Ten individuals were interviewed. Only one individual was  Asian, and the other nine were Caucasian. | Three themes emerged from the data. The first was “choosing life over death,” where participants discussed  how they made a decision to improve their health. The second theme was “learning to live a new self,” where participants  described the changes they had to make in order to improve their health. The third theme was “a life-transforming  cardiac event” where participants shared how the cardiac event had changed their life. |
| Nicolai et al., 2018 | Semi-structured interviews were conducted with 21 individuals who had recently  experienced an AMI. The interviews were audio-recorded and transcribed verbatim. The data  were analysed using qualitative content analysis. | Germany | To investigate key factors related to lifestyle changes  following acute myocardial infarction (AMI) by eliciting survivors’ subjective needs for, attitudes  towards and experiences with behaviour changes in their everyday life to improve future  interventions promoting lifestyle changes. | Cardiac rehabilitation | 21 participants (13 men and 8 women), ranging from 38-79 years of age. | The data revealed that lifestyle changes following AMI are influenced by a  combination of individual (physical and psychological) and social factors that can be grouped into  facilitators and barriers. The interviews indicated the need for more personalised information  regarding the causes and risk factors of illness, the benefits of lifestyle changes and the importance  of including significant others in lifestyle advice and education and of individualising support. |
| Pietrabissa et al,(2015) | Qualitative case study and using interview. | Italy | To integrate theory with practice by describing  a three-session case scenario. | Cardiac rehabilitation | A case was a 39 years-old man, resident in the  south of Italy., at admission to the hospital,  weighing 143 kgs. With an initial BMI of 51.9, calculated by  dividing the weight (in kg) by the square of the height (in meters),  he falls into the highest of the level of obesity (super morbid  his problematic life-style have not been taken into account. The  obesity), presenting increased risk for several health-related conditions.  Giorgio was diagnosed with dilated cardiomyopathy, a  serious condition in which the heart muscle becomes weakened,  therefore unable to satisfy peripheral requirements. He also has a  prosthetic heart valve. | By the use of MI principles and techniques, the patient reported an  increase in his motivation and ability to change, developing a post discharge plan that  incorporates self-care behaviours. |
| White et al (2010) | In-depth qualitative interviews were conducted and audiotaped with 15 patients approximately three  months after hospital discharge, after they had completed a hospital-based cardiac rehabilitation programme.  Repeat interviews with ten patients explored whether their perspectives had changed when interviewed again  approximately nine months later. | England | To understand patients’ perspective on CR lifestyle change and taking medicines. | Cardiac rehabilitation | All 15 patients attended the CR programme ranging from 72-55. They were white British, and 13 were  married and living with their partner. | Patients tended to talk about the exercise component of cardiac rehabilitation and only talk about the  information provision component when prompted, which suggested they viewed the programme as being  primarily about exercise. They seemed to have little subsequent contact with health services, except routine  six-monthly check-ups for their coronary heart disease. Unmet information needs were common, especially  about medicines. Nevertheless, all patients reported continuing to take cardiac medicines, but tended to only  maintain changes to aspects of lifestyle perceived as causes of coronary heart disease, rather than viewing  lifestyle recommendations as standards to achieve. |
| White et al (2011) | Following ethical approval, in-depth, audiotaped, qualitative interviews  were conducted with 15 post-myocardial infarction CR patients (11 men  and four women) who had completed a hospital-based CR programme. Participants  comprised White British individuals aged 42–65 years, from a variety of  socioeconomic backgrounds. Interview topics included perspectives on CHD  and lifestyle changes, including diet. Follow-up interviews with 10 patients,  conducted approximately 9 months later, explored whether their perspectives  had changed | England | To explore CR patients’ perspectives on making and  maintaining dietary changes. | Cardiac rehabilitation | Eleven of the participants were men and four were  women. All were White British individuals aged 42–  65 years, although most patients were aged between 50  and 65 years, and from a variety of socioeconomic backgrounds.  Thirteen patients were married and living with  their partner, two were not living with a partner and  none of the patients were related to each other. | Results: Patients tended to only make and maintain dietary changes if they perceived  their diet to be a cause of their CHD. The only dietary changes patients  reported involved ‘cutting things out’ of their diet; patients did not make dietary  changes if they considered that they did not need to ‘cut things out’. |
| Wong *et al.(2016)* | A qualitative descriptive study design was used, and 22 CHD  patients were recruited in Hong Kong in 2014. In-depth interviews and  content analyses were conducted. The tripartite model of attitudes  was adopted as research framework | Hong Kong | To examine the attitudes of Chinese patients  with coronary heart disease (CHD) toward the outpatient cardiac  rehabilitation program (OCRP), as well as their exercise behaviour,  intention, maintenance and related factors. | Cardiac rehabilitation | 22 participants, 15 (68.2%) were men, and 18 (81.8%) were married patients with a mean age of 54 years ranging from 43 years to 74 years. | Two themes were identified: (1) informant attitude (perception,  affection, and practice) toward the OCRP and (2) Exercise Behavior  – intention, maintenance and its related factors. Most informants  showed positive perception and affection regarding the outpatient  rehabilitation program, leading to regular practice of exercise in  the program and at home. Peer, group dynamic, social support and  Chinese culture influences on exercise behavior may serve as major  facilitators to maintain exercise behavior. |
