# Supplementary material for: The changes in health-related quality of life after attending cardiac rehabilitation: A qualitative systematic review of the perspective of patients living with heart disease
Source: PLoS One. 2025 Jan 30;20(1):e0313612. doi: 10.1371/journal.pone.0313612 (PMC11781667; doi:10.1371/journal.pone.0313612)
Supplement: S7 File — (DOC) [file pone.0313612.s007.DOC]

**Supplementary File 7: Data Extraction**

**Data extraction included studies**

All Data was extracted by the first author.

| **Study** | **Key findings** | **Themes/ categories** |
| --- | --- | --- |
| Clark *et al.* (2005) | Results. Participants’ accounts indicated that the didactic content of cardiac reha-  bilitation was not strongly linked to longer-term health behaviour change. The main positive effects of cardiac rehabilitation were related to the effect of participation on mediating social and body-focused mechanisms that were triggered when the reha- bilitation setting was perceived to be safe. Social mechanisms identified included social comparisons, camaraderie, and social capital. Body-focused mechanisms included greater knowledge of personal physical boundaries and a greater trust in the heart- diseased body. Collectively, these mechanisms had a positive effect on confidence that  was perceived as being imperative to maintain health behaviour change. | **The process of attending CR: the self and the group, the outsider and the insider**  *“*I was wary because I have never been active at all as you can probably see. I have never been particularly ﬁt and I was concerned about doing exercises in front of other people (Pt 4, 5 and 6 agree). In case I made a fool of myself…it has always been a problem with me: We always stayed at the back (referring to self and Pt 5). We stayed in the very, very last row so nobody would watch us”  **The mechanisms of cardiac rehabilitation: how change was promoted and inhibited**  “At the end of the day, you have to get on with your life. You have got to leave the nest. Some people were saying ‘Can I come back?(to CR)’ I can see the social element, but that is not what it is about. You have to be back being independent”.  *“*My whole pattern of life has changed. I had never been to a gym before and I reached 69 years of age and suddenly I have found I am enjoying it and of course, it is geared for my age group and my particular problem. Before you didn’t know your limitations, but after, deﬁnitely after doing rehabilitation you could tell exactly what you could do. And I haven’t looked back since. I don’t do heavy work but, reasonably, I now know there is lots I can do” |
| Dechaine *et al.* (2018) | Both women and men view CR as a means to move forward from their cardiac incident and  achieve good health. Women were more likely than were men to be motivated by a perceived obligation to CR staff and improved quality of life. Men, in comparison, identified their commitment to CR as a need to complete a task. | **Motivations to CR Attendance and Completion**  “I’m looking at it as longevity and the quality of my life. Quality of life to me is key; I don’t want to sit in a wheelchair somewhere where I’m incontinent and I’m contributing nothing. That isn’t living; that’s existing.^ (F12) recovery would mean that I myself would feel very comfortable continuing stuff that I’m doing at the rehab center, like with the exercise, making sure that I’mdoing something every day, and that I don’thavea problem with it… Just to know that I’mgoing to be okay and confident enough to do my exercising every day by myself, you know, onmy own.^ I knew I was on a monitor … and I’dpushit harder, because I figured if I was going to have a heart attack, I might as well have it here when they had me monitored instead of at the gym… I really used cardiac rehab just to reinforce the fact that I could go to the gym and do what I wanted to do, and not have to worry about having a heart attack.  Some of the things I went to when I got home from the nursing home-- I went [to a nursing home] because it would please the kids. The asked me to go, and I really didn’twanttogo, but after I went, I felt better... By them encouraging  me to get out and do stuff other than just going to a doctor’s appointment, it had me looking forward to something, and it got me out of that little funk I was in… If you have somebody that’s encouraging you to do something, you do it just to please them  **Cardiac journey** “Recovery would mean that I myself would feel very comfortable continuing stuff that I’m  doing at the rehab center, like with the exercise, making sure that I’mdoing something every day, and that I don’thavea problem with it… Just to know that I’mgoing to be okay and confident enough to do my  exercising every day by myself, you know, on my own”. |
| Joker *et al.* (2017) | Trying to stay alive and begin again arose as the  two main themes related to behavioural change. These  themes include the changes in cognition and values that  lead to improvements in the process of behavioural change as  a major outcome of cardiac rehabilitation. | **Wanting to change: “**I used to overindulge when eating. At lunchtime, I always ate the fattiest food served at the restaurant. I never took my high cholesterol seriously**”.**  **Finding myself :** I guess no one could ever say that he does not love himself. This is something natural. I can say that I love myself. What I’m saying is that if I can manage to do whatever I’ve been told by my doctors, I mean shed some kilos and lose weight, I can prove that my health matters to me.  **Keeping my heart : “**A month after my operation, I threw a party where I cooked. I did not want any help, so I did all the cooking. By doing so, I also wanted to test myself. I passed. I wanted to feel the difference before and after going under the knife”.  **Living symptoms free : “**I promised to myself that I would not go to work unless I was physically fit and ready to work. I made this pact with myself after starting cardiac rehabilitation”  **Continuation of cardiac rehabilitation** :” have a number I want to reach, let say 100. I am aiming to reach that number before my final session. Next, I would like to try my best to maintain this positive outcome even after I leave here. I would also like to continue doing vigorous exercises, which I have learnt from you and your colleagues, in order to maintain my health” |
| Mead et al. (2010) | We identiﬁed nine major themes reﬂecting issues related to patients’ ability to care for and  manage their heart conditions. We grouped the themes into three domains of interest: (1) barriers that  interfere with getting necessary services, (2) barriers that impede the monitoring and management of a  heart condition on a daily basis, and (3) supports that enable self-management and improve care.  Conclusion: For disadvantaged populations, typical problems associated with self-management of a  heart condition are aggravated by substantial obstacles to accessing care. | **Barriers to accessing necessary services: “**The doctor is hesitant to order the test you need. They don’t even attempt to do it because they assume you can’t pay for it.... Sometimes the lack of insurance does affect your care. ‘The doctor gives you six prescriptions and Medicaid only pays for three, so what happens with the other three? In that case I just don’t buy ‘em”.  **Supports to improving self-management**  ‘**Social support** ‘I have people I can go to and talk to—my brother, my son. I talk to them about everything. All of them  working to support me.’’  ‘‘My wife does all this stuff, like a nurse. That’s her ﬁrst priority. I say, ‘I gotta take 11 pills this morning?’  and she says, ‘I wanna see you alive.’’’  ‘‘Sometimes you just need someone to listen to you. If you feel like crying, just cry. You think you in that  boat by yourself, but all of us are riding in that same boat.’’  **Secondary prevention, disease management programs**  ‘‘Since they referred me back here I had a complete turnaround.... Before I couldn’t even walk to the bathroom.  Now I’m doing well. I can walk over a mile.’’  ‘‘It’s a regimented routine, rather than trying to do it at home. You’d slip otherwise. I would.’’  ‘‘The rehab has been making me feel better. I’m tired in the morning, but when I leave I’m full of energy.’’  ‘‘It’s also the social life. It’s really the center of my life.’’  ‘‘I don’t know what I would have done without [the cardiac rehab staff]. Because when you go home you’re  on your own.  **Psychological issues** It is overwhelming because it’s like a physical and mental thing.... Sometimes I look around and I actually  cry because I can’t handle it... I’m losing control.’’  ‘‘I believe the stress you have in your life can often be more damaging than the cholesterol.’’  ‘‘What you used to do, you can’t do. I used to love to go ﬁshing, hunting swimming. But I can’t do that now.  You get to where you don’t care if you wake up the next morning.’’  ‘‘The meds I take are just too much medicine. I can’t take it anymore.’’  ‘‘I would get so stressed I wouldn’t take my medicine |
| McPhillips et al. (2021) | Results Two main themes were identified: (1) general  therapy factors that were seen largely as beneficial, where  patients highlighted interaction with other CR patients  and CR staff delivery of treatment and their knowledge of  cardiology; (2) group MCT- specific factors that were seen  as beneficial encompassed patients’ understanding of the  intervention and use of particular group MCT techniques.  Most patients viewed MCT in a manner consistent with the  metacognitive model. All the patients who completed group  MCT were positive about it and described self- perceived  changes in their thinking and well- being. A minority of  patients gave specific reasons for not finding the treatment  helpful. | **Interaction with other CR patients : “**Group MCT provided a positive environment where patients felt comfortable to talk about their thoughts, which they may otherwise have kept to themselves: ‘It was good to get it out…you opened up a bit once you started talking… I found that really helpful… they brought things out you’d keep deep down in you”  **CR Staff delivery of group MCT**: I was hung up about standing up like at AA… it wasn’t like that… it was alright because the cardiac rehab nurses I knew anyway, it wasn’t like talking to a stranger… so it wasn’t a big issue talking in the group… they care about you out of therapy anyway… you could speak to them about anything  **Patient experiences of using group MCT techniques**: Postponement, that’s a good thing… you think, ‘oh I can’t deal with that, I’m gonna deal with that next Thursday’… for me that works… you sort of hung that problem on a hook and you move on… and it doesn’t come back into your head I found’  The thought comes into your head and then you let that worry you… they’re teaching you to control that… which I thought was a good thing, but I said to them… when I’ve sorted the house, when we’ve moved… once I’ve sorted my problems out I can see how that [Group- MCT] is gonna be miles more beneficiaL  **Self-perceived changes as a result of group MCT: “**I just feel dead privileged that I can just like draw on it whenever I want… So it’s there and as long as I need it and I want to use it, which I will do… so it is not just six weeks, it’s kind of like life- changing for me” |
| Meredith *et al.* (2019) | Composite  narratives illustrated the emotional intensity of recovering from a cardiac  event and highlighted the value of CR to aid patients with reskilling and  emotional support. In discussing our data, we emphasise the potential value  of emotional intelligent care provision, and the creation of an environment  that encourages emotional disclosure. | **Surviving anxiety: ‘I mean physically I am ﬁne, but mentally I am struggling:** I was fearful, but now I feel like I am managing better. You know, you have a heart attack and they get you in  and put a stent in you and it happens so quick, and then you’re at home again. Then they ask you to exercise,  and you think, “are you CRAZY? I’ve just had a heart attack!” But it’s a supportive environment here, this has  been so useful”. “I just had no idea that anxiety could be so crippling. I mean physically I am ﬁne, but mentally I am struggling. I can’t believe how much the anxiety changes you, even to the extent that I can actually feel tingling down my arms!”I’ve learnt how to control it. I understand that fear is there for a reason, it’s the reason I got blue lighted into hospital, and for a good reason, it allowed me to seek treatment. I just didn’t know how to control it, and  manage it so that it wasn’t detrimental to my health. It got to the point where my anxiety wasn’t saving me  anymore; it was actually putting me in a dark place – not killing me, but deﬁnitely stopping me live  **Putting on a brave face: ‘I think feeling ill is an indication of weakness. It’s like the runt in a litter of dogs, and the weak one doesn’t survive:** He was just so closed! He seemed almost rude and aggressive, he had such a bad attitude. But I think it’s really because of his insecurities and his fear; you know people don’t know what to expect. He’s probably got a lot on his mind. I could see him trying to hold back tears at one point. I see it quite a lot; it’s like the grieving process after a heart attack. At the beginning they’re often in denial –  “this hasn’t happened to me”–and then they start going through the whole, “I can accept it” stage, and then  they’re sad about it, and then they come to the, “I have to deal with it” stage.  **An inspiration: ‘It’s having that sense of humour and positive mind set’**: Yeah I felt the same when I was going through this. It’s like treading on egg shells after you’ve had a heart  attack. I just wasn’t sure how much I could do, you know, you don’t want to break the shells. But coming here  I found that I could do a lot more than I thought.  June shared positively. Yeah exactly, and I am deﬁnitely doing a lot more than I was. I’m not going to let it beat me; I don’t want to be a cardiac cripple! Emotionally it’s very tiring, because the other pains I have are constant. But it’s all about having a sense of humour! |
| Mitchel *et al.* (1999) | Individuals who consistently participated in a cardiac exercise program did not use  strategies to overcome barriers; rather, in their quest to survive,  they used strategies to ensure their participation in the program. | Barriers to participation were refined into three groups. The first group comprised physical limitations. Subjects identified medication side effects, fear of heart transplant rejection, arthritis, and residual effects from a cardiovascular accident as factors that limited the intensity of their exercise. The second group of barriers had to do with sense of self. The subjects' comments included the following: “I'm shy and entering a new group was difficult,” “I'm basically just lazy,” and “I didn't have anything to wear.” The third group of perceived barriers focused on financial concerns. Financial constraints can have a great influence on attendance and adherence with cardiac rehabilitation programs. Two of the subjects were employed full time, one subject was employed part time, and three of the subjects were retired.  The subjects were asked, “What barriers did you overcome in order to participate in the exercise program?” All six subjects responded that they did not have to overcome any barriers. One individual stated, “I'll do whatever I have to do so I never go through another heart attack again.”  **They reported that activity was essential to their good physical and mental health, increased energy level, and improved flexibility**.  **Strategies:** In response to the question, “What have you changed in your life in order to participate in the program?” all subjects indicated that they had to make a lifestyle change after their cardiac event, and joining the exercise class was the easiest change to make. One subject indicated that the cardiologist made the referral to the rehabilitation program (the subject also stated that the doctor did not give him a choice). Other strategies the six identified were using compensation time and changing schedules at work, budgeting money for the class fee each month, and “getting up earlier.”  **Importance of exercise:** The importance of exercise was rated high by all subjects, that people consistently reported a sense of well-being as a direct benefit of engaging in exercise. Each of the six subjects stressed that being active was of key importance to them. They reported that activity was essential to their good physical and mental health, increased energy level, and improved flexibility. One subject reported, “I feel better now than I ever did before.” Another subject reported that she had had 90% coronary artery closure at the time of her MI and “now my heart is normal after 4 years of exercise.”  All six subjects stated they would not exercise on their own if they were given that option. The cardiac exercise class provided the opportunity, not only for physical training, but also for sociability. As one person said, “I've made a lot of friends here and that's what keeps me coming back.” Each subject expressed that the sense of camaraderie they felt in the exercise program was a major motivating factor in their participation.  Subjects were asked what they thought would happen if they stopped exercising. One answered, “I'd rather not contemplate it.” Another individual stated, “I have a sincere appreciation for the individuals who saved my life. I owe them.” The subjects' overall consensus was they would become sedentary, gain weight, and risk having another cardiac event if they did not remain active. |
| Nadarajah *et al.* (2017) | Three themes emerged from the data. The first was “choosing life over death,” where participants discussed  how they made a decision to improve their health. The second theme was “learning to live a new self,” where participants  described the changes they had to make in order to improve their health. The third theme was “a life-transforming  cardiac event” where participants shared how the cardiac event had changed their life. | **Choosing life over death.** The theme “Choosing life over death” describes an increased awareness of mortality that  led individuals to make improving their health a priority. After participants were stabilized medically from their  acute cardiac event, they began processing and reflecting on their experience, ultimately deciding that they wanted  to live. The majority of participants decided to make important changes while they were still in the hospital  after the cardiac event. One participant explained this process by stating “Life. Choosing life over death.  Really, it becomes that simple… Life and death is a really good  **Learning to live a new self:** You see a lot of people who are a lot older than I am who have been doing this for some number of years and that leads you [to] think that, ‘well, of course, everybody dies eventually, but it’s not imminent.’  I can just kind of almost take myself on a little meditation [during a walk in the park] and I went so far as to put an  addition on my house that is all windows so that I can look out into the green because I find greens and blues very calming. After completion of phase two CR, some participants had difficulty overcoming previous lifestyle problems  because of lifestyle barriers, such as inconsistent dieting and exercise. One of the employed men reported, “I’m  pretty good now on other [exercise] metrics but the meeting and the snacks, and the food just kills me. I  don’t have the willpower to push it away.” Most participants participated in exercise programs, for example, but many had difficulty with making healthy diet changes. Some participants were anxious and depressed because  of concern over their inability to exercise, and therefore decided to participate in phase three CR. Almost all persons were worried about having Another cardiac event: “[The] hardest part to Live with… [is] knowi it’s going to happen again.” Most individuals were worried about family security, as One participant explained, “I Have a 22-year-old son, and I have a wife and all that kind of stuff, so you worry about your mortality.” In addition, some worried about finances, specifically about making less money after their cardiac event and spending more money for medications, out-of-pocket phase three CR, and losing life insurance. Despite the negative emotions after their cardiac event, participants adopted a more positive outlook on their lives, regardless of challenging life circumstances. One of the men explained it like this, I think attitude is key. I think it really is important to have a good attitude, and I think that sets the stage for a lot of positives in terms of trying to deal with the challenges that a heart attack or a critical illness might bring.  **A life transforming cardiac event.** But, a lot of this is also, you know, post-heart attack. I use it. People say, “What are you doing?” I said, ‘I am doing what I However, not think is important.’ You know, and it is like, okay, they [my work administrator] are afraid to push me. So, I use it … You can always blame your heart. Then they back off, Oh yeah, yeah. ‘Your health comes first.’ Because I went on this diet, I have an excuse to say to some of my friends [at a previous job], ‘I don’t want to go to lunch every week.’ I go to exercise Monday, Wednesday, Friday, and I am in here [CR] from 11 am to 1 pm. Some of the things I was doing with them were not stimulating. They were just time consuming. Whereas it was like – this sort of gave me an excuse to back out of some of those relationships or to keep  them down real low. |
| Nicolai et al., 2018 | The data revealed that lifestyle changes following AMI are influenced by a  combination of individual (physical and psychological) and social factors that can be grouped into  facilitators and barriers. The interviews indicated the need for more personalised information  regarding the causes and risk factors of illness, the benefits of lifestyle changes and the importance  of including significant others in lifestyle advice and education and of individualising support. | acilitator: Positive physical feedback and Barrier:  Physical impairments and comorbidities. Most of  the interviewees stated that they monitored  physiological processes and that positive  physical feedback led to increased selfconﬁdence  and increased motivation to  maintain changes. However, physical  impairments and comorbidities were often  mentioned as limiting factors and barriers to  lifestyle changes, particularly with respect to  physical activity. Those interviewees who  reported considerable physical impairments  and comorbidities often mentioned lifestyle  changes in the manner of ‘I would like  to ...but I can’t’  **Psychological factors**:All the participants indicated  that they were surprised by the diagnosis  of a heart attack. Most of the  interviewees described being in shock after  hearing the diagnosis and felt they ﬁrstneeded to address the complex emotions  (i.e., happiness regarding survival, sadness,  anxiety) that the diagnosis evoked. After  that, many of the participants expressed that  they engaged in a self-reﬂection process  aimed at ﬁnding meaning in the AMI.  Viewing their heart attack as a chronic  condition enhanced the interviewees’ readiness  to implement lifestyle changes. Some of  the participants experienced having a heart  attack as a ‘warning shot’ and as receiving a  second chance. Accordingly, the participants  who reported lifestyle changes noted  that they reappraised their life domains,  reviewed their lives and re-evaluated and  changed their priorities.  **Self-motivation and self-discipline:** have a heart rate monitor, and I always look for it when I get out of breath, so that I regulate breathing again using the breathing technique and take a break when I notice that it doesn’t work, when I notice that I can’t breathe anymore, simply taking a break  **Facilitator: Social support.** Support from family, friends and neighbours was frequently cited as beneﬁcial to our interviewees’ eﬀorts to adopt healthy lifestyles. Most of the interviewees stressed the importance of instrumental support from family and friends. Emotional support from family and friends was also mentioned as a source of  comfort. It ranged from the mere presence of family and friends via emotional counselling to mutual activities. This support was important because it conveyed feelings of safety and facilitated a return to normalcy. Because recently fears came up once again (...) And knowing that I am not alone is giving me a sense of security.  Most of the participants indicated that stress was a major factor in having a heart attack. However, the CR attendees stated that stress-reduction programmes were frequently misdirected and therefore not transferrable  to their daily life. Simply to get of that image... the image that smokers are themselves to blame if they There are so many situations (...) where I get excited, ﬂy into a rage, where everything gets out of control. But I can’t come along, position myself and do yoga or stuﬀ like that. |
| Pietrabissa et al,(2015) | By the use of MI principles and techniques, the patient reported an  increase in his motivation and ability to change, developing a post discharge plan that  incorporates self-care behaviours. | Lack of physical activity and poor diet had led him to progressive weight gain and experiencing signiﬁcant daily life diﬃculties. Giorgio was perfectly aware that his behavior was counter-productive for his health status, but he stated he simply could not do otherwise. Reﬂectively listening, the therapist started focusing on engaging the patient, building interest in change, and eliciting willingness to implement self-care behaviors. have gradually gained weight not realizing it and now... 4 months ago, I have started to think about everything. And I have to change.” (Change talk Need).  Rather than focusing on the negative experiences of his failure When a person is ambivalent about changing behavior, it is in following the care plan, the practitioner continued exploring Giorgio’s goals and values. Apart from his own health status, his main motivation for changing were his wife and three children. o get back to a healthier status and spend more time with my children.  During the previous week, something had changed within the patient’s mind. In fact, for the first time, he felt the need to completely open up to the therapist, revealing the real and intimate reason that has led him to stop taking care of himself. Giorgio had never verbally expressed it before, with an obvious liberating effect. The basic emotion sustaining the problem is the fear, to which an important guilt is linked (“I’m afraid of getting back to being fit, then eventually relapsing back into the problem”).  The encounter proceeded exploring any additional worries of the patient. Despite presenting realistic concerns in mind regarding his health status, he expressed sincere need for well-being and clear goals. Regarding his health objectives, he formulated specific plans of action and showed realistic understanding of the time necessary to achieve lasting results (“I’m hopeful... I give myself a year and a half … I do not believe it would be enough time to get to the point I would like to reach. However, I do hope to get to the point of being 80% independent”).  I was wondering, when a person continues to implement behaviors he knows being harmful for himself, for his health... There must be a reason.... Otherwise it’s torturous, a self-destruction, isn’t it? Must be some motives that lead you to act in this way. What do you think?” […]  “Look, I hope to continue following the diet at home … and regarding my previous conduct, looking back … it was just a sort of self-destruction! That’s it!” |
| White et al (2010) | Patients tended to talk about the exercise component of cardiac rehabilitation and only talk about the  information provision component when prompted, which suggested they viewed the programme as being  primarily about exercise. They seemed to have little subsequent contact with health services, except routine  six-monthly check-ups for their coronary heart disease. Unmet information needs were common, especially  about medicines. Nevertheless, all patients reported continuing to take cardiac medicines, but tended to only  maintain changes to aspects of lifestyle perceived as causes of coronary heart disease, rather than viewing  lifestyle recommendations as standards to achieve. | **Perspectives on the CR programme**: .before I started to go on the rehab, exercise thing [CR programme]  I suppose it was a fear in the back of your mind that you were frightened of doing something that’ll bring it on again. People I’ve had dealings with in the rehab group have always made it clear that they would be happy to see me again and talk things over with me if I ever needed to and the door has always been left open, which is nice. It’s reassuring  when people are like that but I haven’t taken them up on it. The people I’ve had dealings with in the rehab group have always made it clear that they would be happy to see me again and talk things over with me if I ever needed to and the door has always been left open, which is nice. It’s reassuring when people are like that but I haven’t taken them up on it.  **Perspectives on information received**: She said keep fat as low as possible. Well, you know, that was all the help we had. I mean we’ve been to the library and got low fat cookery books and things like that, but you don’t know whether you’re doing right or wrong.  **Perspectives on lifestyle modiﬁcation**: You carry on your lifestyle, your body’s saying look I’m not happy I’ve got a problem, if you don’t address the problem it will happen again won’t it...I want to make sure I don’t have another one. You carry on your lifestyle, your body’s saying look I’m not happy I’ve got a problem, if you don’t address the problem it will happen again won’t it...I want to make sure I don’t have another one.  **Perspectives on information received**: She said keep fat as low as possible. Well, you know, that was all the help we had. I mean we’ve been to the library and got low fat cookery books and things like that, but you don’t know whether you’re doing right or wrong. |
| White et al (2011) | Results: Patients tended to only make and maintain dietary changes if they perceived  their diet to be a cause of their CHD. The only dietary changes patients  reported involved ‘cutting things out’ of their diet; patients did not make dietary  changes if they considered that they did not need to ‘cut things out’. | **Cutting things out’**: making dietary changes, most patients explicitly talked about dietary changes they had made but only in terms of ‘cutting things out’ of their diet, and appeared to perceive that their diet had been a cause of their heart attack. For example, one patient reported ‘trying to cut out fatty foods’, one patient said that he had ‘certainly cut down on a lot of things’, whereas another said: ‘I’m cutting things out, puddings and sugary things, fatty things so I’m being more careful with my diet … You carry on your lifestyle, your body’s saying look I’m not happy I’ve got a problem, if you don’t address the problem it will happen again won’t it … I want to make sure I don’t have another one.’ (R15: male) One patient talked about reducing his ‘fat intake a lot’ to reduce his cholesterol, which he perceived to be a cause of his heart attack. Two patients talked about speciﬁc foods they had ‘cut out’ of their diet but in terms of being ‘bad’ foods or foods they ‘can’t have’. One of these patients talked about enjoying ‘soft cheese and things that are bad for me’ and added, ‘now I only eat it occasionally, not much’. She said ‘it was a contributory factor that  I smoked and I’m overweight’, whereas the other patient said: ‘… I didn’t realise what damage some of the stuff were doing to you … I love cheese and I love fryups. I don’t have them any more. I don’t have sugar … Chocolate I can’t have and … Oh yes, and ﬁsh and chips  **Maintaining dietary changes** In the follow-up interviews, some patients appeared to have maintained the dietary changes that they said they had made by the time of the initial interview. For example, one patient said ‘I really do watch what I eat whereas another said that, as a result of the changes he had made, ‘we don’t really have a bad diet, we don’t eat fat stuff’. In addition, another patient (who only implied that he had changed his diet in the initial interview) said that he had lost ‘over a stone’ as a result of an ‘enforced salad diet’ and had ‘cut a lot of stuff out’. ‘I got another episode in my leg [of cellulitis] and I thought you can’t sort of sit and not eat anything!’  **Not making dietary changes**: not needing to ‘cut things out’ By contrast, in the initial interviews, two patients explained why they had not made dietary changes but did so in terms of not needing to ‘cut things out’ of their diet, and did not perceive that their diet had been a cause of their heart attack. One of these patients said, ‘diet has never been a problem because I always enjoy what happens to be good for me’, whereas the other patient said: I don’t think my diet is all that bad; I’ve not been to McDonalds or had takeaways … I have very little cooked in fat or lard or grease or anything like that … I’m not exactly overweight … I don’t stuff myself with fried egg and bacon and things like that. I have them occasionally but I don’t eat them on a daily basis. I don’t eat things like beef burgers or stuff like that. |
| Wong *et al.(2016)* | Two themes were identified: (1) informant attitude (perception,  affection, and practice) toward the OCRP and (2) Exercise Behavior  – intention, maintenance and its related factors. Most informants  showed positive perception and affection regarding the outpatient  rehabilitation program, leading to regular practice of exercise in  the program and at home. Peer, group dynamic, social support and  Chinese culture influences on exercise behavior may serve as major  facilitators to maintain exercise behavior. | Perception toward OCRP differed among CHD patients. All informants generally regarded OCRP as an essential part of the therapeutic schedule. They expected that exercise schedule embedded in OCRP was useful for their recovery, and regular exercise would be a positive health behavior that they should keep doing even upon OCRP completion. On the one hand, participants claimed that they should keep the exercise habit as prescribed in the OCRP. On the other hand, 41% claimed that they may not have time to exercise as scheduled because of work commitment. Two informants related their experience as follows: The OCRP was very useful, and I lost some weight and felt stronger in my leg muscles. Exercise is good for my health, especially for my heart … I think it can help me to resume my health and I can go to work again after completion of the program …. (Informant 2 who completed the OCRP) As I expected, the OCRP was useful and trained my arm and leg muscle stronger. However, I start to regain my work gradually from next week and therefore I may miss one or two sessions per week in the remaining class. Anyway, I tried my best to attend all if possible …. (Informant 5 who had completed half of the OCRP class) Affection associated with OCRP CHD patients shared positive and affirmative affections toward OCRP. In general, all the CHD patients strongly agreed that the role of OCRP was aligned with their personal xpectations that the rehabilitation class can assist them to resume an improved life as normal as possible or because they expressed feelings of wanting to take good care of their  health. An informant shared her experience as follows: I liked OCRP exercise. It built up my exercise habit. In the CR center, I feel very safe for taking exercise. The nurses and physiotherapist can guide me and provide support if I feel discomfort during exercise. The content of CR program is useful for daily life such as stress management  and diet advises … I hope that I can resume my usual activities as before …. (Informant 10) OCRP class is important to me and I want to complete it in order to strengthen my body again. I want to resume my normal work again …. (Informant 14 whose age was 50 years old and completed PCI two months ago) Some of the CHD patients were motivated to perform more OCRP exercises, with one saying: I am not very diligent with exercising. In the OCRP, doctors motivated me a lot to exercise even though I felt a bit tired after that … I think I will complete the class …. (Informant 6) OCRP practice CHD patients exhibited various experiences of practicing OCRP. Followed the guidelines, whereas 41% (n = 9) created their own CR plans and only attended selected sessions. The good practices of CHD patients included adherence to the OCRP organized by rehabilitation centers, participation in a variety of lectures related to CR and development of individual CR plans according to comprehensive assessments conducted by nurses, therapists and social workers. One informant summarized the affection toward OCRP and ways for further maintenance of exercise behavior as follows: I tried to search for ways to support my exercise habit. Luckily, the CR center nurse suggests a plan for me, in which I could set my goals, achieve my goal and record my achievements. The CR center provides an easy-to-follow schedule of exercise, so I keep on following the assigned tasks. Eventually, I could form a habit of walking exercise daily …. (Informant 5) Theme 2: Exercise behavior intention, maintenance, and its related factors We analyzed the patients’ responses regarding their beliefs related to factors affecting their intention and maintenance for exercise habit to determine how they maintain their behavioral habits. Informants shared their experience about the factors affecting exercise behavior intention and maintenance. Peer, group dynamic and social support  In this study, support from family, friends and colleagues was found to be important for CHD patients in maintaining exercise behavior. Five patients (23%) mentioned that exercise together with group dynamic was particularly important for long-term exercise engage- ment. In addition, four participants (18%) claimed that their spouses supported them well through verbal reminder and even accompanied them to join the daily exercises. Three selected informants related their experience as follows: Exercise peers in OCRP are important for me to keep joining until completion of the OCRP class. I like to meet them every week … Sometimes, if I feel lazy, my friends will remind me to keep it up. Now we always meet and join some other low level hiking activities together … Walking together is fun and we can talk about everything when we walk …. (Informant 20)  My boss is very kind to me. He helped me change the job nature and offered me sufficient time to exercise after work. At the beginning, I am a bit worry of my rehabilitation program will disturb my work, but my colleagues showed understanding and empathy … I am happy that I don’t create much problem in my work due to my health problem … I want to keep working to support my family. (Informant 8)  My family is very supportive … My wife encourages me a lot. She reminds me to adhere to healthy diet and exercise maintenance. She walks with me every day …. (Informant 9 who completed the OCRP class 1 month ago) |
