# Supplementary material for: The changes in health-related quality of life after attending cardiac rehabilitation: A qualitative systematic review of the perspective of patients living with heart disease
Source: PLoS One. 2025 Jan 30;20(1):e0313612. doi: 10.1371/journal.pone.0313612 (PMC11781667; doi:10.1371/journal.pone.0313612)
Supplement: S8 File — (DOC) [file pone.0313612.s008.DOC]

**Supplementary File 8:** **Themes and Categories**

| Synthesised Finding | | | |
| --- | --- | --- | --- |
| Studies | Physical health | Social functioning | Mental and emotional health |
| Clark *et al.* (2005) | 🗸 | 🗸 | 🗸 |
| Dechaine *et al.* (2018) |  |  |  |
| Joker *et al.* (2017) | 🗸 |  | 🗸 |
| Mead et al. (2010) |  |  | 🗸 |
| McPhillips et al. (2021) |  |  | 🗸 |
| Meredith *et al.* (2019) |  | 🗸 | 🗸 |
| Mitchel *et al.* (1999) | 🗸 | 🗸 |  |
| Nadarajah *et al.* (2017) | 🗸 |  |  |
| Nicolai et al., 2018 | 🗸 |  |  |
| Pietrabissa et al,(2015 |  |  | 🗸 |
| White et al (2011) | 🗸 |  |  |
| White et al (2010) | 🗸 |  |  |
| Wong *et al.(2016)* | 🗸 | 🗸 |  |
